# Rétinopathie diabétique proliférante: stade redoutable évoluant vers la cécité

**DOI:** 10.11604/pamj.2014.17.124.3985

**Published:** 2014-02-21

**Authors:** Salim Belhassan, Rajae Daoudi

**Affiliations:** 1 Université Mohammed V Souissi, Service d'Ophtalmologie A de l'hôpital des Spécialités, Centre Hospitalier Universitaire, Rabat, Maroc

**Keywords:** Rétinopathie diabétique, diabéte, décollement de rétine, diabetic retinopathy, diabetes, retinal detachment

## Image en medicine

Patiente âgé de 31 ans, originaire d'un milieu rurale et défavorisé ayant comme antécédent: diabétique type I mal suivie découvert lors d'un dépistage de masse par une association dans son village et monophtalme de l'oeil gauche depuis 10 ans. Accuse une baisse de l'acuité visuelle depuis plus de 5 ans au niveau de l'oeil gauche, l'examen actuelle trouve une acuité visuelle a 1/20, le segment antérieur est sans particularité (bonne chambre antérieure, cornée clair, iris de trame et de coloration normale, absence de rubéose irienne, cristallin clair) le tonus oculaire était à 15 mmHg. Le fond d'oeil trouve quelques impacts de laser en périphérie et surtout en temporal inférieure et une membrane proliférative prenant la totalité du côté nasal arrivant et prenant attache sur la papille avec extension vers les arcades vasculaire supérieure et inférieure temporales. La rétinopathie diabétique proliférante a des conséquences graves sur le pronostic visuel, le diagnostic précoce est la clé du traitement de la rétinopathie diabétique à un stade non encore proliférative. Dans le présent cas où la rétinopathie diabétique est très évoluée le traitement de choix est la panphotocoagulation rétinienne pour éliminer toute zone d'ischémie et de néovaisceaux et d’éviter les complications à type de décollement de rétine tractionnelle ou d'hémorragie intra vitréenne.

**Figure 1:**
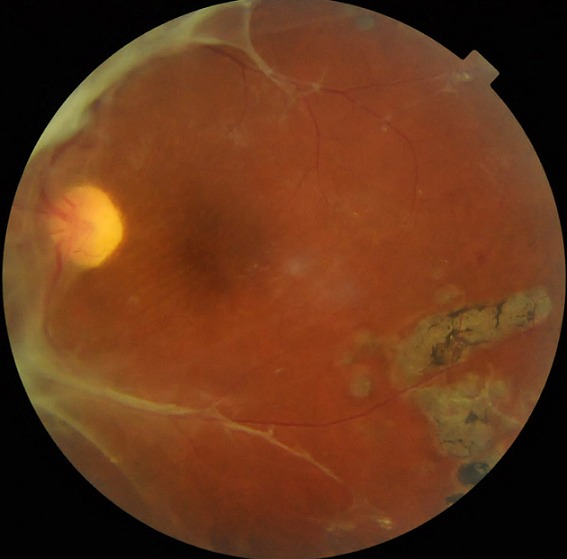
Image caractéristique de la rétinopathie diabétique montrant la membrane proliférative siégeant au côté nasal et prenant fixation sur la papille avec extension vers les deux arcades vasculaires temporales supérieure et inférieure

